# Pai syndrome: a review

**DOI:** 10.1007/s00381-020-04788-z

**Published:** 2020-07-10

**Authors:** Francesca Olivero, Thomas Foiadelli, Sabino Luzzi, Gian Luigi Marseglia, Salvatore Savasta

**Affiliations:** 1 Department of Pediatrics, Fondazione IRCCS Policlinico San Matteo, Pavia, Italy; 2 Department of Clinical, Surgical, Diagnostic and Pediatric Sciences, University of Pavia, Pavia, Italy; 3 Neurosurgery Unit, Department of Surgical Sciences, Fondazione IRCCS Policlinico San Matteo, Pavia, Italy

**Keywords:** Pai syndrome, Frontonasal dysplasia, Cleft lip, Nasal polyp, Facial polyp, Corpus callosum lipoma

## Abstract

**Background:**

Pai syndrome is a rare idiopathic developmental condition characterized by midline craniofacial abnormalities. It was originally described as the presence of a median cleft lip, cutaneous polyps of the nasal mucosa and face, and midline lipomas of the central nervous system, mostly at the corpus callosum. However, there is great phenotypical variability and these characteristics are rarely all present at once.

**Objective:**

The aim of this review was to analyze the available evidence regarding Pai syndrome in order to better delineate this rare condition and its features.

**Methods:**

We analyzed the PubMed database using the words “Pai syndrome”, “frontonasal dysplasia”, “cleft lip”, “nasal polyp”, “facial polyp”, and “corpus callosum lipoma”, including reviews, case reports and case series.

**Conclusion:**

There is no consensus regarding the diagnostic criteria of Pai syndrome up to date. It is usually diagnosed at birth, and its incidence is often underestimated. At present, the etiology of Pai syndrome is unknown. Several hypotheses regarding its genetic background have been made; however, there are not enough data yet to elucidate this point. An improved awareness could help in diagnosing the condition and performing the necessary investigations. These patients should have a multidisciplinary follow-up.

## Introduction

Pai syndrome is a rare condition, defined as a syndromic form of frontonasal dysplasia [1, 2]. It was first described by Pai et al. as “an unusual combination of three rare developmental anomalies: complete median cleft lip, cutaneous polyps, and midline lipomas of the central nervous system” [2]. It has a high phenotypical variability, and most of the cases described in literature do not meet the full triad of criteria originally defined by Pai, which is a relatively restrictive definition [3]. About sixty cases of Pai syndrome have been described in literature up to date. Among them, only 19 patients met the full triad of criteria originally described by Pai, while the majority showed great phenotypical variability [3].

In 2019, Morice et al. defined Pai syndrome as the association of a congenital nasal and/or mediofrontal skin mass and/or a mid-anterior alveolar process polyp as a mandatory criterion, and at least one other criterion: midline cleft lip and/or midline alveolar cleft, and/or a pericallosal lipoma or interhemispheric lipoma in the case of corpus callosum dysgenesis [3].

In 2007, Castori et al. defined Pai syndrome as the presence of at least two criteria among: one or more hamartomatous nasal polyp(s) and the presence of a midline facial cleft and/or mid-anterior alveolar process congenital polyp [4].

A more inclusive definition was given by Lederer et al. who defined Pai syndrome as the presence of a congenital nasal polyp plus one of the following three feature: midline cleft lip, mid-anterior alveolar process congenital polyp, or a lipoma of the corpus callosum [5].

Overall, the most inclusive definition is the one given by Morice et al. where 91.6% of the patients matched the diagnostic criteria. However, there is still no consensus on the definition of Pai syndrome.

Pai syndrome has no gender preponderance [3, 6]. To our knowledge, no risk factors have been described for Pai syndrome, as a better understanding of this condition and its pathophysiology is necessary. At present, its etiology is unknown [3, 7].

Lees et al. described a family with five generations affected by midline cleft lips and nasal dermoids, possibly supporting the autosomal dominant inheritance pattern theory [8, 9]. Pai syndrome has also been hypothesized to have an X-linked recessive inheritance, as its incidence has been reported to be higher in males than in females in the past [6]; this has not been confirmed in recent reviews and case series [3]. A case of a 13-year-old Japanese girl with clinical features of Pai syndrome has been reported by Masuno et al. in 1997. The girl was shown to have a “de novo*”* reciprocal translocation 46,X,t(X;16)(q28,q11.2), hypothesized to be candidate regions for median cleft of the upper lip and pedunculated facial skin masses, therefore suggesting that cytogenetic studies might have a role in other patients with these characteristics, including those with Pai syndrome [6]. The phenotype described by Masuno et al. overlaps with frontonasal dysplasia (FND), as it is often observed in Pai syndrome, that has been defined as a syndromic form of FND [1, 2]. When it was performed, chromosomal analysis has been shown to be normal in some previous case reports of Pai syndrome [10, 11]; however, in patients with additional features, chromosomal abnormalities might be present (in particular when overlapping with FND or other conditions of the oculo-auriculo-frontonasal spectrum.

The pathogenesis of midline abnormalities derives from disruptions during embryological development: the midline lies at a structural nexus that unites individual elements, in a fusion process. Defects at this point can result in a diverse range of phenotypes and clinical entities that are included in the FND spectrum [12]. The embryological development of the lip starts at the 4th week of gestation [13]. Two theories regarding the pathogenesis of median facial clefts have been described: the classical theory states that the growth, meeting, and fusion in the midline of the lateral maxillary processes and the unpaired frontal process medially are responsible for normal facial development [8, 11]. Veau instead asserts that a deficient mesodermal penetration in the primary palate would cause a median lip cleft [1, 11].

With our literature review our aim was to analyze the available evidence regarding Pai syndrome in order to better delineate this rare condition and its features. We analyzed the PubMed database using the words “Pai syndrome,” “frontonasal dysplasia,” “cleft lip,” “nasal polyp,” “facial polyp,” and “corpus callosum lipoma.” We selected scientific papers from 1980 to 2019 including reviews, case reports, and case series.

## Clinical features

**Fig. 1:**
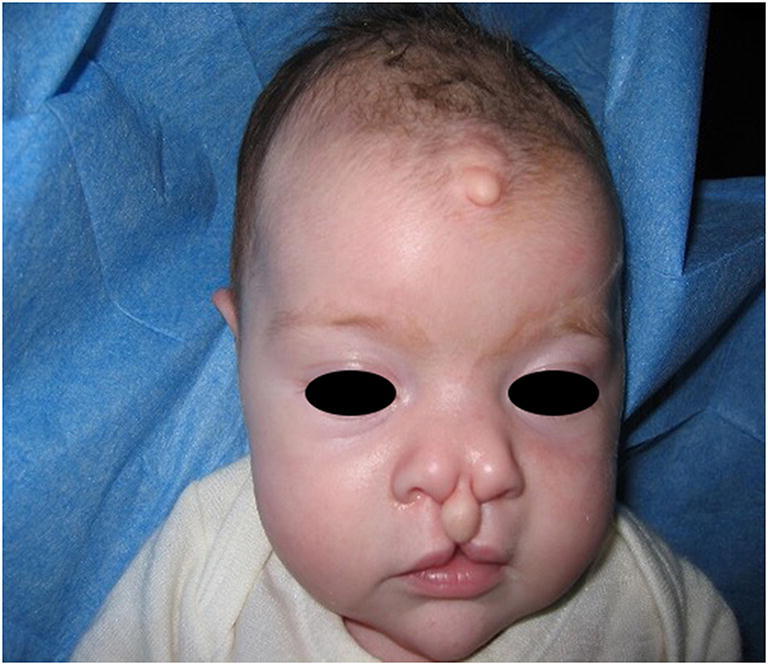
Facies of a patient with Pai syndrome [7]

In Pai syndrome, midline clefting is variable and there are mild forms involving the teeth (i.e., isolated diastemas of the maxillary dentition), and severe forms extending to the upper lip, the surrounding structures, and the nose [1, 7] (Fig. 1).

Furthermore, some authors suggested a relationship between the degree of clefting and the cranial involvement [1, 14]. In patients affected by Pai syndrome, a slight separation of the wing of the nose has been reported; other anomalies of the midline spectrum such as divided frenulum of the upper lip, bifid uvula, high palate, and midline sinus have also been reported [1, 7]. Among midline anomalies, cleft lip represents one of the most common ones, with an incidence of 1:1000 [13]. However, midline cleft lip is rare and constitutes 0.43 to 0.73% of cases of cleft lip and palate [3, 7, 11].

Pai syndrome is also associated with various type of congenital nasal and facial anomalies involving the anterior skull base and meninges, caused by a maldevelopment of the embryonic mesenchyme, the latter having a paramount role in reparative processes of the central nervous system [15, 16].

Facial hamartomatous polyps of the midline have been shown to be a typical feature of Pai syndrome, mostly at the nasal structures, as originally described by Pai [2], and lesions associated with this syndrome have to be differentiated from other pathologies involving the nose, nasal cavity, and nasopharynx, as the sphenoidal and sincipital cephalocele, nasal dermal sinus cyst, and nasal glioma [14, 17, 18].

The persistence of the fonticulus frontalis or pre-nasal space beyond the eighth week of gestation is responsible for a delayed ossification of the “crista galli” and cribriform plate and is related to the occurrence of cephaloceles and naso-facial lipomas, both potentially associated with syndromic frontonasal dysplasia (FND), as well as Pai syndrome [1, 3, 7, 14, 19]. The generic term of cephaloceles also includes meningoencephaloceles and meningoceles, which in the context of Pai syndrome, generally involve the midline facial skeleton and the nasal cavity. Cephaloceles are of two types, namely, frontoethmoidal and skull base. Frontoethmoidal, or sincipital, type involves the herniation of the brain tissue into forehead, dorsum of the nose, or orbit. Within the sincipital group, frontonasal, naso-ethmoidal, and naso-orbital variant has been reported to have an incidence of 60%, 30%, and 10%, respectively [19, 20]. On computerized tomography (CT) scan, nasal cephaloceles are well demarcated, heterogeneous, and have a mixed density, whereas they are isointense, show a direct contiguity with basal cortex on magnetic resonance imaging (MRI), and do not enhance after gadolinium, as opposed to certain types of primary brain tumors [19–21].

Nasal lipomas and dermoid cysts frequently present calcifications on CT scan and are homogeneously hyperintense on T1-weighted MRI in a non-negligible number of cases. Nasal lipomas are benign masses that can be usually excised for cosmetic rather than clinical reasons, and this is usually performed in the context of the surgical correction of other midline facial defects [1]. Furthermore, lipomas classically become hypointense with fat suppression [19].

Nasal cerebral heterotopia, also known as nasal glioma, has no connection with intracranial contents and, being a hamartomatous lesion, involves MRI features and molecular mechanisms totally different from those reported for brain gliomas [1, 3].

Congenital intracranial lipomas are rare and comprise 0.1–0.5% of all primary brain tumors. Pericallosal location is the most common (50%) and is usually associated with callosal abnormalities such as dysgenesis or agenesis [22]. In Pai syndrome, central nervous system (CNS) lipomas always involve the corpus callosum; however, other CNS locations have been included by Pai (i.e., spinal cord) in his original description of the syndrome [1, 2]. Pericallosal lipomas represent 0.06–0.46% of prenatally diagnosed intracranial lesions, and they can be detected prenatally by careful ultrasound examination, and MRI, which shows characteristic findings on T2-weighted sequences [23]. Whether they should be considered as malformative lesions rather than tumors is still argument of discussion. CNS lipomas are thought to derive from an over-proliferation of the fat cells of the leptomeninges, and two types have been reported. The most common is the tubulonodular one, usually located anteriorly and associated with extensive callosal and frontonasal anomalies, in particular, agenesis or dysgenesis of the corpus callosum with a less favorable prognosis, while the curvilinear lipoma is posteriorly located and usually thinner than the tubulonodular type [19, 23]. When pericallosal lipomas are isolated they are usually benign and remain asymptomatic, with a good prognosis [22]. Patients with Pai syndrome with a pericallosal lipoma usually have normal psychological development, usually without epilepsy, contrarily to other complex syndromes [24–26], if there are not associated chromosomal abnormalities, and other callosal anomalies [1, 3, 7, 27].

Other features of Pai syndrome include hypertelorism, epicanthus, other ocular anomalies, clinodactyly, cryptorchidism, and inguinal hernia [1, 3, 7]. We have summarized the main other features that were observed in affected patients in Table 1 by analyzing Pai syndrome case reports.

**Table 1:** Additional clinical features in Pai syndrome

Abnormality	Description	References
Ocular	Hypertelorism, palpebral fissure abnormalities, eyebrows abnormalities, epicanthus, epibulbar dermoid, conjunctival lipoma, lacrimal duct abnormality, and micropthalmia	Rudnik-Schoneborn (1994), Masuno (1997), Castori (2007), Guion-Almeida (2007 and 2009), Chousta (2008), Savasta (2008), Vaccarella (2008), Lederer (2012), Tormey (2017), Huckstadt (2018), Zanetta (2011), Mishima (1999), Ocak (2013), and Ferreira Moreno (2016)
Facial + Oral	Bifid nose, broad nasal root or bridge, alae nasi abnormalities, abnormal frontal hairline, wide forehead, bifid labial frenulum, nasal dimple, and skin tags	Coban (2003), Lees (2006), Guion-Almeida (2007), Chousta (2008), Savasta (2008), Ocak (2013), Blouet (2014), Mishima (1999), Castori (2007), Vaccarella (2008), Lederer (2012), and Melloni Magnelli (2015)
Neurological	Corpus callosum hypoplasia or agenesis, and frontal encephalocele	Guion-Almeida (2007 and 2009), Abdelmaaboud (2012), Dobrocky (2015), Huckstadt (2018), Castori (2007), Zanetta (2011), Blouet (2014), and Ferreira Moreno (2016)
Auricular	Enlarged earlobe(s), preauricular fibrochondroma, prominent antihelix and antitragus, and small concha	Castori (2007), Ocak (2013), and Huckstadt (2018)
Genital/Urinary	Cryptorchidism and hypospadia	Pai (1987) and Lederer (2012)
Heart	Interventricular communication and ventricular septal defect	Huckstadt (2018)
Genetics	De novo reciprocal translocation 46,X,t(X;16)(q28,q11.2) and duplication at 4q35.2	Masuno (1997) and Li and Galvin (2018)
Others	Clinodactyly of the 5th digit, aplasia cutis, inguinal hernia, and sacral dimple	Pai (1987), Lederer (2012), Zanetta (2011)

References: [1, 2, 4–10, 17, 23, 28–39]

## Diagnosis

There is no consensus regarding diagnostic criteria for Pai syndrome up to date. This rare syndrome is usually diagnosed at birth [23], and its incidence is underestimated; therefore, an improved awareness could also help in diagnosing the condition and performing the necessary investigations (i.e., brain imaging) to better define the disease phenotype [1].

**Fig. 2:**
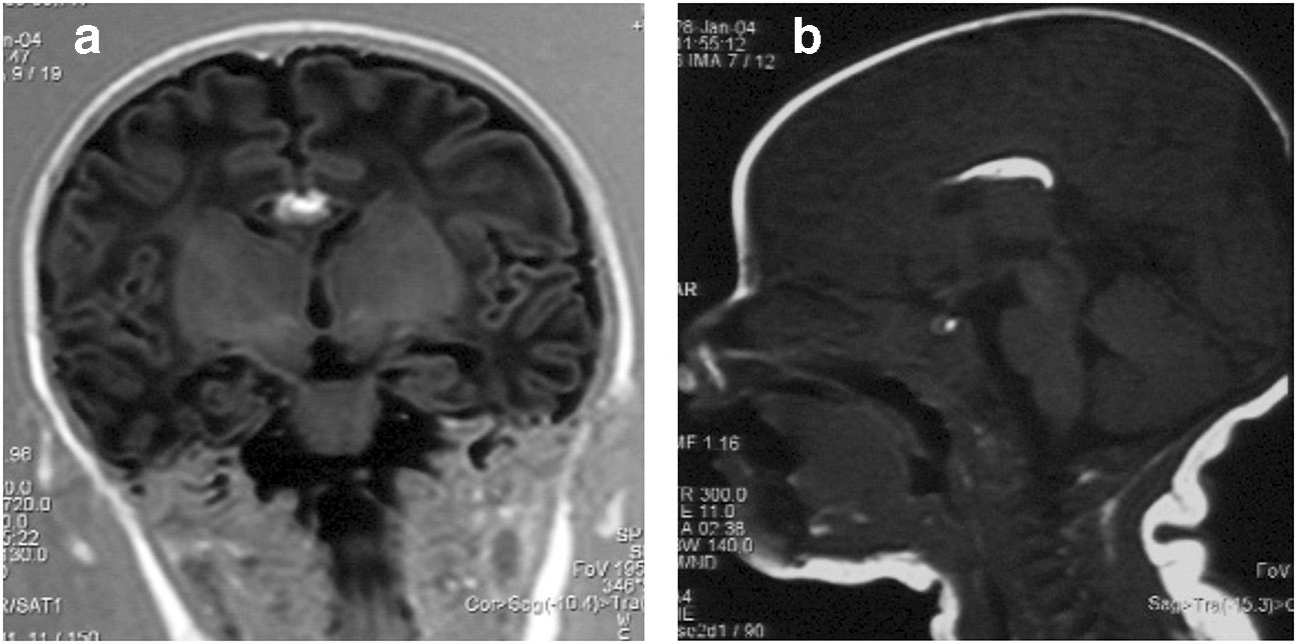
CORONAL and SAGITTAL views of brain MRI: lipoma as a thick hyper-intense band and partial agenesis of the corpus callosum [7]

**Fig. 3:**
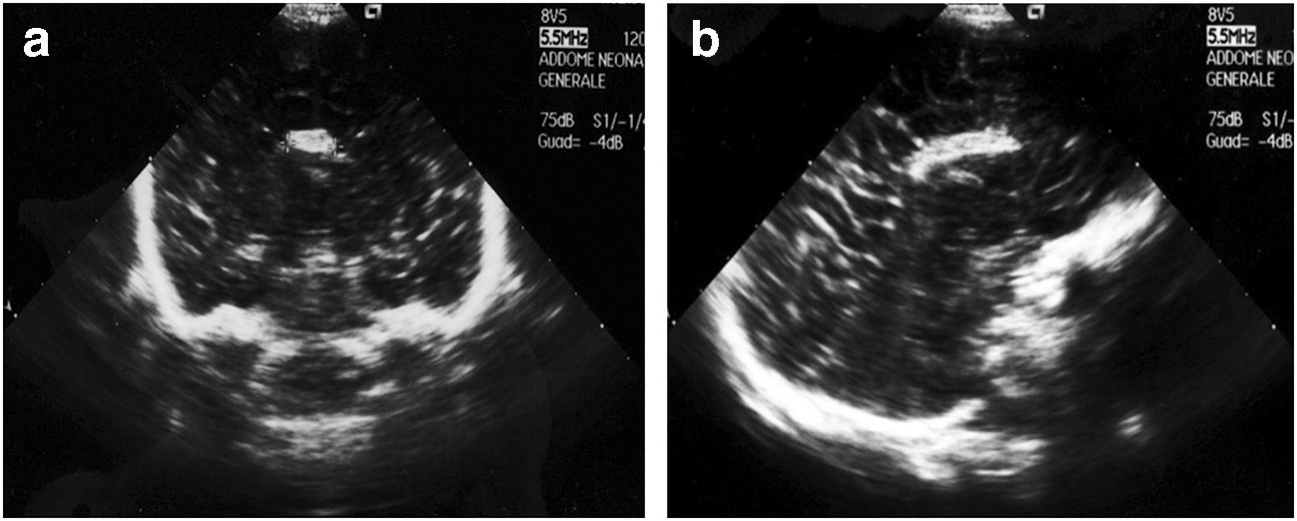
Cranial ultrasound in coronal and sagittal views: agenesis of the corpus callosum and a callosal lipoma, appearing as an interhemispheric midline echogenic mass [7]

Some cases have been suspected by ultrasound examination in the course of pregnancy, and prenatal diagnosis was made by some authors [23, 28]. The usefulness of fetal MRI as a second-level investigation has also been supported for a more complete evaluation of the fetus’ shape and face [23]. A pericallosal lipoma appears as a hypointense signal on T2-weighted sequences because of its fatty content, and it has a hyperintense signal on T1-weighted sequences without fat suppression **(**Figs. 2 and 3**)**. MRI is useful also to define the lesion’s dimensions and associated anomalies of the corpus callosum [23]. Shinar et al. have recently published a paper regarding the clues to diagnose pericallosal lipoma prenatally [40].

## Treatment and prognosis

Surgical correction of midline clefts of the upper lip and removal of midline polyps can be done at once, and it has to meet both functional and cosmetic requirements, restoring a normal appearance and reestablishing the orbicularis oris muscle cohesion. Cheiloplasty is usually performed between 3 and 6 months of age. Correction of the nasal pyramids is usually postponed after puberty in order to allow the growth of the nasal bones [1].

Surgery of the skull base cephaloceles provides for a wide spectrum of transcranial or combined transcranial-transnasal approaches already reported by our group for other cranial neurosurgical pathologies [41–47] and involves an intradural resection of the herniated brain tissue, along with a duraplasty which should be watertight as far as possible [20, 48–50].

Pericallosal lipomas may sometimes become symptomatic later in life causing epilepsy; therefore, regular follow-up is crucial [1, 3, 29, 51]. However, in Pai syndrome they rarely do so [1]. Nevertheless, differently from cephaloceles, intracranial lipomas are universally considered as “leave me alone” lesions by the reasons of their extremely rare symptomatic features, as well as the fact that they always encase brain vessels and nerves, making therefore surgery very dangerous.

Regarding neuro-psychological development, only two patients with Pai syndrome were described as being affected by mental retardation, to our knowledge: the one described by Masuno, who seemed to suffer from a more complex spectrum than Pai syndrome alone, and a patient described in a South American case series [6, 30].

## Conclusion

Pai syndrome is a rare condition characterized by wide phenotypical variability. An improved awareness could help in diagnosing the condition and performing the necessary investigations. In particular, systematic brain imaging should be performed when a congenital midline facial skin mass or cleft is present, together with genetic counseling and genetic investigations. Because some patients showed ocular abnormalities, ophthalmologic examination is also justified [3, 31]. These patients need to be followed up in their physical and neuropsychological development, and this ideally requires a multidisciplinary approach with surgeons (maxillofacial, neurosurgeons, plastics, ENT), the pediatrician and pediatric neurologist, and a geneticist.

## References

[1] Vaccarella F, Pini Prato A, Fasciolo A, Pisano M, Carlini C, Seymndi PL (2008). Phenotypic variability of Pai syndrome: report of two patients and review of the literature. Int J Oral Maxillofac Surg.

[2] Pai GS, Levkoff AH, Leithiser RE (1987). Median cleft of the upper lip associated with lipomas of the central nervous system and cutaneous polyps. Am J Med Genet.

[3] Morice A, Galliani E, Amiel J, Rachwalski M, Neiva C, Thauvin-Robinet C, Vazquez MP, Picard A, Kadlub N (2019). Diagnostic criteria in Pai syndrome: results of a case series and a literature review. Int J Oral Maxillofac Surg.

[4] Castori M, Rinaldi R, Bianchi A, Caponetti A, Assumma M, Grammatico P (2007). Pai syndrome: first patient with agenesis of the corpus callosum and literature review. Birth Defects Res A Clin Mol Teratol.

[5] Lederer D, Wilson B, Lefesvre P, Poorten VV, Kirkham N, Mitra D, Verellen-Dumoulin C, Devriendt K (2012). Atypical findings in three patients with Pai syndrome and literature review. Am J Med Genet.

[6] Masuno M, Imaizumi K, Fukushima Y (1997). Median cleft of the upper lip and pedunculated skin masses associated with de novo reciprocal translocation 46, X t(X;16) (q28; q11.2). J Med Genet.

[7] Savasta S, Chiapedi S, Perrini S, Tognato E, Corsano L, Chiara A (2008). Pai syndrome: a further report of a case with bifid nose, lipoma and agenesis of the corpus callosum. Childs Nerv Syst.

[8] Rudnik-Schoneborn S, Zerres K (1994). A further patient with Pai syndrome with autosomal dominant inheritance?. J Med Genet.

[9] Lees MM, Connely F, Kangesu L, Sommerlad B, Barnicoat A (2006). Midline cleft lip and nasal dermoids over five generations: a distinct entity or autosomal dominant Pai syndrome?. Clin Dysmorphol.

[10] Coban YK, Boran C, Omeroglu SA, Okur E (2003). Pai syndrome: an adult patient with bifid nose and frontal hairline marker. Cleft Palate Craniofac J.

[11] Szeto C, Tewfik TL, Jewer D, Rideout A (2005). Pai syndrome (median cleft palate, cutaneous nasal polyp, and midline lipoma of the corpus callosum): a case report and literature review. Int J Pediatr Othorhinolaryngol.

[12] Farlie PG, Baker NL, Patrick Y, Tiong YT (2016). Frontonasal dysplasia: towards an understanding of molecular and developmental aetiology. Mol Syndromol.

[13] Worley ML, Patel KG, Kilpatrick LA (2018). Cleft Lip and Palate. Clin Perinatol.

[14] Al-Mazrou KA, Al-Rekabi A, Alorainy IA, Al-Kharfi T, Al-Serhani AM (2001). Pai syndrome: a report of a case and review of the literature. Int J Pediatr Othorhinolaryngol.

[15] Luzzi S, Crovace AM, Del Maestro M, Giotta Lucifero A, Elbabaa SK, Cinque B, Palumbo P, Lombardi F, Cimini A, Cifone MG, Crovace A, Galzio R (2019). The cell-based approach in neurosurgery: ongoing trends and future perspectives. Heliyon.

[16] Luzzi S, Crovace AM, Lacitignola L, Valentini V, Francioso E, Rossi G, Invernici G, Galzio RJ, Crovace A (2018). Engraftment, neuroglial transdifferentiation and behavioral recovery after complete spinal cord transection in rats. Surg Neurol Int.

[17] Guion-Almeida ML, Richieri Costa A (2009). Frontonasal dysplasia, severe neuropsychological delay and midline central nervous system anomalies: report of 10 Brazilian male patients. Am J Med Genet A.

[18] Rodriguez DP, Orscheln ES, Koch BL (2017). Masses of the nose, nasal cavity, and n in children. Radiographics.

[19] Salzman AGOGHKL (2017). Osborn's brain.

[20] Kabré A, Zabsonre DS, Sanou A, Bako Y (2015). The cephaloceles: a clinical, epidemiological and therapeutic study of 50 cases. Neurochirurgie.

[21] Luzzi S, Elia A, Del Maestro M, Elbabaa SK, Carnevale S, Guerrini F, Caulo M, Morbini P, Galzio R (2019). Dysembryoplastic neuroepithelial tumors: what you need to know. World Neurosurg.

[22] Yadav A, Kumar J (2018, 2018) Pericallosal lipoma in children: a rare case. BMJ Case Rep 2018 Mar 1;2018:bcr2017223989 10.1136/bcr-2017-223989 PMC5847960 29496688

[23] Blouet M, Belloy F, Jeanne-Pasquier C, Leporrier N, Benoist G (2014). Pai syndrome: challenging prenatal diagnosis and management. Pediatr Radiol.

[24] Matricardi S, Darra F, Spalice A, Basti C, Fontana E, Dalla Bernardina B, Elia M, Giordano L, Accorsi P, Cusmai R, De Liso P, Romeo A, Ragona F, Granata T, Concolino D, Carotenuto M, Pavone P, Pruna D, Striano P, Savasta S, Verrotti A (2018). Electroclinical findings and long-term outcomes in epileptic patients with inv dup (15). Acta Neurol Scand.

[25] Ivanovski I, Djuric O, Caraffi SG, Santodirocco D, Pollazzon M, Rosato S, Cordelli DM, Abdalla E, Accorsi P, Adam MP, Ajmone PF, Badura-Stronka M, Baldo C, Baldi M, Bayat A, Bigoni S, Bonvicini F, Breckpot J, Callewaert B, Cocchi G, Cuturilo A, De Brasi D, Devriendt K, Dinulos MB, Hjortshøj TD, Epifanio R, Faravelli F, Fiumara A, Formisano D, Giordano L, Grasso M, Grønborg S, Iodice A, Iughetti L, Kuburovic V, Kutkowska-Kazmierczak A, Lacombe D, Lo Rizzo C, Luchetti A, Malbora B, Mammi I, Mari F, Montorsi G, Moutton S, Møller RS, Muschke P, Nielsen JEK, Obersztyn E, Pantaleoni C, Pellicciari A, Pisanti MA, Prpic I, Poch-Olive ML, Raviglione F, Renieri A, Ricci E, Rivieri F, Santen GW, Savasta S, Scarano G, Schanze I, Selicorni A, Silengo M, Smigiel R, Spaccini L, Sorge G, Szczaluba K, Tarani L, Tone LG, Toutain A, Trimouille A, Valera ET, Vergano SS, Zanotta N, Zenker M, Conidi A, Zollino M, Rauch A, Zweier C, Garavelli L (2018). Phenotype and genotype of 87 patients with Mowat-Wilson syndrome and recommendations for care. Genet Med.

[26] Verrotti A, Cusmai R, Laino D, Carotenuto M, Esposito M, Falsaperla R, Margari L, Rizzo R, Savasta S, Grosso S, Striano P, Belcastro V, Franzoni E, Curatolo P, Giordano L, Freri E, Matricardi S, Pruna D, Toldo I, Tozzi E, Lobefalo L, Operto F, Altobelli E, Chiarelli F, Spalice A (2015). Long-term outcome of epilepsy in patients with Prader-Willi syndrome. J Neurol.

[27] Pascual-Castroviejo I, Pascual-Pascual SI, Perez Higueras A (1985). Fronto-nasal dysplasia and lipoma of the corpus callosum. Eur J Pediatr.

[28] Chousta A, Ville D, James I, Foray P, Bisch C, Depardon P, Rudigoz RC, Guibaud L (2008). Pericallosal lipoma associated with Pai syndrome: prenatal imaging findings. Ultrasound Obstet Gynecol.

[29] Mishima K, Mori Y, Minami K, Sakuda M, Sugahara T (1999). A case of Pai syndrome. Plast Reconstr Surg.

[30] Guion-Almeida ML, Mellado C, Beltrán C, Richieri-Costa A (2007). Pai syndrome: report of seven south American patients. Am J Med Genet A.

[31] Li E, Galvin JA (2018). Ophtalmic abnormalities of Pai syndrome: a case report and review of literature. Ophtalmic Genet.

[32] Tormey P, Bilic Cace I, Boyle MA (2017). Ocular dermoid in Pai syndrome: a review. Eur J Med Genet.

[33] Huckstadt V, Heis Mendoza ME, Moresco A, Obregon MG (2018). Pai syndrome: two new cases with unusual manifestations. Arch Argent Pediatr.

[34] Zanetta A, Cuestas G, Oviedo M, Tiscorni C (2011). Unilateral nasal obstruction in children: Pai syndrome. Arch Argent Pediatr.

[35] Ocak Z, Yazicioglu HF, Aygun M, Ilter MK, Ozlu T (2013). Prenatal detection of Pai syndrome without cleft lip and palate: a case report. Genet Couns.

[36] Ferreira Moreno VG, Sosa Fundora I, Domínguez Boffill S, Vidal Tallet LA, Orea Cordero I, Alonso Gálvez C (2016). Pai syndrome without cleft lip. A variation in the expression of the syndrome: a case report. J Med Res.

[37] Melloni-Magnelli LF, de la Garza-Giacomán R, Martínez-Leija H, Guzmán-Rodríguez R (2015) Primer caso clínico de Síndrome de Pai en México. Cirugía Plástica Ibero-Latinoamericana 41:183–189

[38] Abdelmaaboud M, Nimeri N (2012) Pai syndrome: first reported case in Qatar and review of literature of previously published cases. BMJ Case Rep. 2012 Aug 21;2012:bcr0220125940 10.1136/bcr-02-2012-5940 PMC4543044 22914230

[39] Dobrocky T, Ebner L, Liniger B, Weisstanner C, Stranzinger E (2015). Pre- and postnatal imaging of Pai syndrome with spontaneous intrauterine closure of a frontal cephalocele. Pediatr Radiol.

[40] Shinar S, Lerman-Sagie T, Telleria ME, Viñals F, García R, Quiroga H, Bermejo C, Ben-Sira L, Leibovitz Z, Har-Toov J, Malinger G (2018). Fetal pericallosal lipomas—clues to diagnosis in the second trimester. Eur J Paediatr Neurol.

[41] Arnaout MM, Luzzi S, Galzio R, Aziz K (2020). Supraorbital keyhole approach: pure endoscopic and endoscope-assisted perspective. Clin Neurol Neurosurg.

[42] Gallieni M, Del Maestro M, Luzzi S, Trovarelli D, Ricci A, Galzio R (2018). Endoscope-assisted microneurosurgery for intracranial aneurysms: operative technique, reliability, and feasibility based on 14 years of personal experience. Acta Neurochir Suppl.

[43] Luzzi S, Gallieni M, Del Maestro M, Trovarelli D, Ricci A, Galzio R (2018). Giant and very large intracranial aneurysms: surgical strategies and special issues. Acta Neurochir Suppl.

[44] Luzzi S, Giotta Lucifero A, Del Maestro M, Marfia G, Navone SE, Baldoncini M, Nunez M, Campero A, Elbabaa SK, Galzio R (2019). Anterolateral approach for retrostyloid superior parapharyngeal space Schwannomas involving the jugular foramen area: a 20-year experience. World Neurosurg.

[45] Luzzi S, Maestro MD, Elia A, Vincitorio F, Perna GD, Zenga F, Garbossa D, Elbabaa SK, Galzio R (2019). Morphometric and radiomorphometric study of the correlation between the foramen magnum region and the anterior and posterolateral approaches to ventral intradural lesions. Turk Neurosurg.

[46] Luzzi S, Zoia C, Rampini AD, Elia A, Del Maestro M, Carnevale S, Morbini P, Galzio R (2019). Lateral Transorbital Neuroendoscopic approach for Intraconal meningioma of the orbital apex: technical nuances and literature review. World Neurosurg.

[47] Zoia C, Bongetta D, Dorelli G, Luzzi S, Maestro MD, Galzio RJ (2019). Transnasal endoscopic removal of a retrochiasmatic cavernoma: a case report and review of literature. Surg Neurol Int.

[48] Songür E, Mutluer S, Gürler T, Bilkay U, Görken C, Güner U, Celik N (1999). Management of frontoethmoidal (sincipital) encephalocele. J Craniofac Surg.

[49] Wu X, Zhang J, Zhang H, Xie Z, Fan R, Liu Y, Wu B, Sun H, Jiang W (2016). Endoscopic surgery for congenital basal meningoencephaloceles in children. Acta Otolaryngol.

[50] Zabsonre DS, Kabre A, Haro Y (2015). Frontoethmoidal cephalocele: our experience of eleven cases managed surgically. Pediatr Neurosurg.

[51] Gastaut H, Regis H, Gastaut JL (1980). Lipomas of the corpus callosum and epilepsy. Neurology.

